# Editorial: Current trends in targeted and non-targeted metabolomics in analytical toxicology

**DOI:** 10.3389/fmolb.2025.1614399

**Published:** 2025-06-04

**Authors:** Geraldine M. Dowling, Markus R. Meyer

**Affiliations:** ^1^ Department of Life Sciences, School of Science, Atlantic Technological University, Sligo, Ireland; ^2^ Cameron Forensic Medical Sciences, William Harvey Research Institutes, Barts and The London School of Medicine and Dentistry, Queen Mary University of London, London, United Kingdom; ^3^ Department of Analytical, Environmental and Forensic Sciences, School of Cancer and Pharmaceutical Sciences, Faculty of Life Sciences and Medicine, King’s College London, London, United Kingdom; ^4^ Experimental and Clinical Toxicology and Pharmacology, Center for Molecular Signaling (PZMS), PharmaScienceHub (PSH), Saarland University, Homburg, Germany

**Keywords:** analytical toxicology, personalised medicine, metabolomics, interdisciplinary approach, medicine

Analytical toxicology is at the frontline of detecting, identifying, and quantifying xenobiotics, drugs, and their metabolites in biological specimens. This essential discipline intersects with several fields, including analytical and clinical chemistry, pharmacology, and environmental health. Particularly in personalised medicine, analytical toxicology plays an essential role as metabolomics, analyzing small molecules in biological systems, plays a pivotal role in this evolution. By enabling both targeted and untargeted analyses, metabolomics allows for rapid screening of metabolites, helping to identify changes in physiological states triggered by toxic exposure or drug interactions. This approach not only enhances the precision of toxicological testing but also broadens the scope of detection, allowing for a deeper understanding of how substances affect human health on a molecular level (Zhu et al.; Barla et al.). This allows the integration of metabolomics into personalised medicine, opening up new frontiers in both research (Mojsak et al.) and clinical applications. What makes metabolomics particularly pioneering in the context of personalized medicine is its ability to capture the individual biochemical responses to drugs or toxins. Every person’s metabolic profile differs, influencing how they process and react to substances. By incorporating metabolomics into analytical toxicology, we can better understand these unique individual responses, paving the way for tailored therapeutic strategies. This strategy allows clinicians to move away from one-size-fits-all treatments, instead offering interventions that are finely tuned to each patient’s specific metabolic makeup. In turn, this can improve patient outcomes by optimizing drug efficacy and reducing adverse effects. Furthermore, metabolomics allows for more precise monitoring of ongoing drug use or exposure, offering the option of real-time adjustments to treatment plans. In the case of substance misuse, metabolomics could help track metabolites over time, enabling more effective management strategies, personalized detoxification protocols and faster recovery timelines. This flexibility is essential for addressing the evolving nature of drug misuse and ensuring that healthcare approaches are not only reactive but proactive.

As the landscape of drug misuse also continues to evolve, the importance of advanced analytical methods in toxicology testing has never been greater. Fundamentally, analytical toxicology serves a critical function in ensuring the safety and wellbeing of individuals by providing accurate testing for drug misuse, environmental toxins, and other harmful substances. However, the escalating complexity of substances involved in drug abuse and toxic exposure demands persistent innovation in analytical techniques. The struggle lies in keeping up with new drugs, metabolites, and their potentially changeable effects on the human body. For this reason, joint communication and collaboration between clinicians, legal experts, law enforcement, and toxicologists are essential to effectively address these issues.

The combination of analytical toxicology, metabolomics, and personalized medicine is poised to revolutionize the way we approach drug misuse, exposure, and treatment. This interdisciplinary approach promises better detection, more precise diagnoses, and customized therapeutic strategies that improve both health outcomes and public safety. As research in these fields continues to evolve, so too will our ability to provide smarter, more effective solutions for patients and society as a whole.

This editorial Research Topic highlights how the integration of metabolomics into analytical toxicology offers fresh perspectives. As personalized medicine continues to gain momentum, metabolomics stands at the forefront of tailoring healthcare to the individual. By offering deeper insights into how specific drugs and toxins affect each person, we move closer to precision medicine—where treatments are based on a comprehensive understanding of an individual’s metabolic and biochemical makeup.

Zhu et al. employed mass spectrometry-based metabolomics to investigate the effects and underlying mechanisms of isochlorogenic acid A in MC3T3-E1 cells. Their findings offer valuable insights into the therapeutic potential of 3,5-DiCQA for osteoporosis and demonstrate the effectiveness of metabolomics in advancing the understanding of traditional Chinese medicine (TCM).

In their study, Mojsak et al. applied gas chromatography–mass spectrometry (GC-MS)-based metabolomics to investigate how metabolite profiles change post-mortem in porcine blood, comparing samples collected with and without EDTA anticoagulant. Using linear mixed models, they examined how metabolite levels were influenced by time since death and the presence of EDTA, while also accounting for variability between individual animals. Their results revealed that 16 metabolites—primarily amino acids—were significantly affected by both post-mortem interval (PMI) and anticoagulant use. The authors emphasized that for a biomarker to be reliable in estimating PMI, its concentration should be driven solely by the time elapsed after death, without being impacted by external factors such as EDTA.

Barla et al. investigated the biochemical disruptions in the kidneys and liver of mice treated with a clinically relevant dose of colistin. Their analysis identified six metabolites (including PAA, DA4S, and 2,8-DHA) that responded in a dose-dependent manner, along with notable disturbances in renal dopamine regulation and significant alterations in purine metabolism within the kidneys. Additionally, the researchers observed changes in hepatic suberylglycine levels—a metabolite associated with fatty liver disease. Elevated concentrations of xanthine and uric acid were also detected in kidney tissue, both known to enhance acetylcholinesterase (AChE) activity, which in turn accelerates the breakdown of acetylcholine. These findings support a simplified hypothesis suggesting a possible link between colistin methanesulfonate (CMS)-induced kidney toxicity and its potential to cause neurotoxic effects—an association that warrants deeper investigation.

Isaiah et al. conducted a study examining the urinary metabolic profile of children in South Africa with advanced tuberculous meningitis (TBM). Their analysis revealed a distinct set of 29 urinary metabolites associated with advanced stages of the disease. These metabolites were linked to six major disruptions in metabolic function: (1) enhanced breakdown of tryptophan, indicating interference with vitamin B pathways; (2) abnormalities in amino acid metabolism; (3) a surge in energy production consistent with a metabolic burst; (4) imbalances in gut microbiota-related metabolism; (5) signs of ketoacidosis; and (6) elevated nitrogen elimination. This work offers novel biological insights into a urinary metabolic signature that may help distinguish paediatric TBM cases within this regional population.

Moses et al. outlined the potential of ion mobility spectrometry in the context of untargeted metabolomics. In this review, the authors compare ion mobility-based separation with liquid chromatography, trace the evolution of ion mobility techniques within metabolomics, present the current advancements and methodologies, and offer perspectives on future developments in the field.

Wang et al. investigated the therapeutic effects and underlying mechanisms of YiYiFuZi powder (YYFZ) in the context of chronic heart disease (CHD), employing both metabolomics and network pharmacology approaches. YYFZ is a traditional Chinese medicinal formula frequently used in clinical practice to manage CHD, though its precise pharmacological actions remain insufficiently understood. Using UPLC-Q-TOF/MS, the researchers conducted metabolomic profiling of rat plasma to identify biomarkers and explore affected metabolic pathways. Additionally, network pharmacology was applied to uncover key molecular targets and signaling pathways involved in YYFZ’s effects. The metabolomic analysis revealed 19 metabolites associated with pathways including amino acid and fatty acid metabolism. The network analysis indicated that YYFZ may exert its effects via the PI3K/Akt, MAPK, and Ras signaling pathways. In conclusion, YYFZ appears to influence systemic metabolism and activate multiple phosphorylation-related signaling cascades in CHD; however, more research is needed to clarify which specific changes are critical to its therapeutic action.

Su et al. conducted a comprehensive investigation involving untargeted serum metabolomics and whole-body fat assessment using dual-energy X-ray absorptiometry (DXA) in a cohort of 517 Chinese women. The study examined four DXA-derived body fat (BF) traits simultaneously to uncover shared metabolite associations and highlight key metabolic contributors. Using a pathway topology approach, the researchers identified biological processes closely linked to body fat regulation. The analysis was further extended by evaluating how these candidate BF-associated metabolites relate to fat traits across different sexes and ethnic groups using two independent validation cohorts. Among the findings, acetylglycine emerged as a standout metabolite, showing strong anti-obesity properties confirmed *in vivo* using multiple models of diet-induced obesity (DIO) in mice. In total, 18 metabolites and 14 metabolic pathways were significantly associated with BF traits, with six metabolites validated across populations of different gender and ethnicity. The consistent, cross-species efficacy of acetylglycine underscores its potential as a therapeutic agent for obesity prevention. Overall, this study highlights the metabolic underpinnings of fat distribution and the biological mechanisms that may influence obesity risk and its management, pointing to acetylglycine as a promising target for future interventions.

Wandy et al. conducted a study comparing data acquisition strategies in untargeted metabolomics by evaluating how well simulated results reflect real-world performance. They enhanced the Virtual Metabolomics Mass Spectrometer (ViMMS) platform by integrating a module for Data-Independent Acquisition (DIA), enabling a detailed *in silico* comparison between DIA and Data-Dependent Acquisition (DDA) approaches. Their findings revealed that method performance is highly dependent on the number of ions eluting simultaneously. When few compounds overlap, DIA delivers superior results; however, as ion overlap increases, DDA proves more effective since DIA struggles to resolve the complexity of densely overlapping chromatographic signals. These simulation outcomes were corroborated using a physical mass spectrometer, confirming that ViMMS simulations can reliably predict real experimental behavior. A major strength of this study lies in ViMMS’s ability to flexibly model and test various parameters across both acquisition modes. This approach not only enhances understanding of DIA and DDA performance but also significantly reduces the need for extensive laboratory testing, offering a powerful tool for advancing LC-MS/MS method development in metabolomics research.

Ji et al. explored how Tongdu Huoxue Decoction (THD) influences the biological network involved in lumbar spinal stenosis (LSS) through a clinical metabolomics approach. Patients were assessed both before and after treatment using the Visual Analogue Scale (VAS) and Japanese Orthopaedic Association (JOA) scores to evaluate pain and lumbar function. Serum levels of Interleukin-1β (IL-1β), Tumor Necrosis Factor-alpha (TNF-α), and Prostaglandin E2 (PGE2) were measured pre- and post-treatment using ELISA assays. Additionally, targeted metabolomic profiling was performed on serum samples from patients (both before and after treatment) as well as from healthy individuals, using Ultra Performance Liquid Chromatography (UPLC). Multivariate statistical analysis was then applied to identify significant changes in metabolites and disrupted metabolic pathways. The clinical findings showed that THD effectively alleviates pain, enhances lumbar function, and reduces inflammatory markers in LSS patients. Mechanistically, these therapeutic effects appear to involve the modulation of purine metabolism, steroid hormone synthesis, and amino acid metabolism-related biomarkers.

